# Research on an Improved Medical Image Enhancement Algorithm Based on P-M Model

**DOI:** 10.2174/1874120701509010209

**Published:** 2015-08-31

**Authors:** Beibei Dong, Jingjing Yang, Shangfu Hao, Xiao Zhang

**Affiliations:** The College of Information Science and Engineering, Hebei North University, Zhangjiakou, Hebei, 075000, China

**Keywords:** Bending, distortion, image enhancement, matlab, P-M model, tilt

## Abstract

Image enhancement can improve the detail of the image and so as to achieve the purpose of the identification of the image. At present, the image enhancement is widely used in medical images, which can help doctor’s diagnosis. IEABPM (Image Enhancement Algorithm Based on P-M Model) is one of the most common image enhancement algorithms. However, it may cause the lost of the texture details and other features. To solve the problems, this paper proposes an IIEABPM (Improved Image Enhancement Algorithm Based on P-M Model). Simulation demonstrates that IIEABPM can effectively solve the problems of IEABPM, and improve image clarity, image contrast, and image brightness.

## 1. INTRODUCTION

In the field of image process, image enhancement is a very important research direction, which has been widely used in military, remote sense, public safety, biomedicine, etc [1]. In the field of medicine, the image is usually gathered by CT machine, ultrasonic apparatus, and so on. The collected images may be blurry, which will affect diagnoses of the illness. To improve the quality of the collected images, image enhancement is used [2]. 

From the aspect of the realization, the image enhancement algorithm can be divided into five categories: image enhancement algorithm base on traditional theory, image enhancement algorithm based on multiscale analysis [3], image enhancement algorithm based on fuzzy theory [4], image enhancement algorithm based on humanoid vision [5] and image enhancement algorithm based on mathematic morphology [6]. Among them, the image enhancement algorithm based on mathematic morphology consists of closing operation, erosion operation, dilation operation and open operation. IEABPM (Image Enhancement Algorithm Based on P-M Model) is one of the most used [7, 8]. IEABPM can effectively remove the noise of images, however, for the area which has rich textures, it may it may cause the lost of the texture details and other features. To solve the problem, IIEABPM (Improved Image Enhancement Algorithm Based on P-M Model) is proposed. And Simulation demonstrates that IIEABPM can improve image clarity, image contrast, entropy and iamge brightness.

## 2. IIEABPM

In IIEABPM, it firstly uses the normalization method to translate P-M model into posed problem. Secondly, a moderator is added to control the process of optimization. Thirdly, according to different region status of the image, IIEABPM chooses the spread function. To meet the performance requirements, the spread function is corrected through four steps. The first step is increasing gradient threshold. The second step is modifying spread function. The third step is adding gradient fidelity term. The last step is adding strength coefficient. Fig. (**1**) gives the flow chart of IIEABPM.

According to Fig. (**1**), when the normalization is used, Gaussian kernel is adopted to smooth the images.

(1) $$u_{t}=div(c\left(\left|\nabla u_{\sigma }\right|)\nabla u\right)$$

Where $u_{\sigma }(x,y,t)=G_{\sigma }*U(x,y,t),andG_{\sigma }$is a Gaussian function, whose mean value and variance are respectively 0 and $\sigma^{2}$.

After the normalization, P-M model is translated into posed problem, and there is only one continuous solution, which depends on the initial value $u_{0}(x,y)$. And then a moderator is added to control the process of optimization, and$u(x,0)=u_{0}(x)\to u(x,y,0)=u_{0}(x,y)$. Therefore, the mathematical model of image enhancement can be written as follows:

(2) $$\begin{array}{l} \frac{\partial u_{t}(x,y)}{\partial t}=div(g\left(\nabla u_{t}\right)\nabla u_{t})\\ u(x,y,0)=u_{0}(x,y) \end{array}$$

Where *u* is the image at time* t*, *div *means divergence operator, $\nabla u_{t}$ is the gradient of the image, $g\left(\nabla u_{t}\right)$is the spread fuction, whose value means the strength of spread. İn some area of the image, the value of $\left|\nabla u_{t}\right|$decides whether or not the image is smooth. That is to say, When the value of $\left|\nabla u_{t}\right|$is smaller, the image in the region is smoother.

In order to keep the anisotropic diffusion of spread function, $\nabla u_{t}$ must make spread function satisfy the following two conditions:

(1)        The spread of the noise is within the relatively smooth featured area;

(2)        The spread is not done between two adjacent areas to preserve the edge details.

When removing the noise of the image, spread function can be chosen from two kinds of expressions:

(3) $$g(\left|\nabla u_{t}\right|)=\frac{1}{1+{\left(\frac{\left|\nabla u_{t}\right|}{k}\right)}^{2}}$$

(4) $$g(\left|\nabla u_{t}\right|)=e^{-{\left(\frac{\left|\nabla u_{t}\right|}{k}\right)}^{2}}$$

### 2.1 The First Step

Although the above spread model has good effects on the image denoise, its effect isn’t ideal when the noise is high. To solve the problem, Eq. 3 and Eq.4 can be modified:

(5) $$g(\left|\nabla u_{t}\right|)=\frac{k_{3}}{1+{\left(\frac{\left|\nabla u_{t}\right|}{k_{1}+k_{2}}\right)}^{2}}$$

(6) $$g(\left|\nabla u_{t}\right|)=e^{-{\left(\frac{\left|\nabla u_{t}\right|k_{3}}{k_{1}+k_{2}}\right)}^{2}}$$

Where $k_{1},k_{2},k_{3}$are the gradient thresholds , and $k_{1}\approx k_{3 ,}k_{1},k_{3}>k_{2}$. Fig. (**2**) gives the relationships between spread function and gradient.

From Fig. (**2**), we can see that the gradient and spread function has an inverse relationship. And compared with Eq. (3) and Eq. (4), Eq. (5) and Eq. (6) can better reserve the edge and texture.

### 2.2 The Second Step

In order to more accurately control the smoothness, $g(\left|\nabla u_{t}\right|)$is replaced by$g\left(\left|\nabla G_{{}_{\sigma }}*u\right|\right)$ , where the expression of $G_{\sigma }$is:

(7) $$G_{\sigma }=\frac{c}{\sqrt{\sigma }}\exp\left(-\frac{x^{2}+y^{2}}{4\sigma }\right)$$

Therefore, divergence operator can be written as:

(8) $$\frac{\partial u}{\partial t}=div\left(g\left(\left|\nabla G_{\sigma }*u\right|\right)\left|\nabla u\right|\right)where,g(x,y,t)=\exp\left(-{\left(\frac{\nabla G_{\sigma }*u}{k}\right)}^{2}\right)$$

When calculating $\nabla G_{\sigma }*u_{0}$, the similarity functional of two signals is:

(9) $$E(u)=\int_{\Omega }a{\left(u-u_{0}\right)}^{2}+\beta {\left(\left|\left|\nabla u-\nabla (\nabla G_{\sigma }*u_{0})\right|\right|\right)}^{2}dxdy$$

Where α and β are weight coefficients,

${\left(\left|\left|\nabla u-\nabla (\nabla G_{\sigma }*u_{0})\right|\right|\right)}^{2}$

is the gradient fidelity term, which try to make the changes of the gradient keep consistent with $\nabla (G_{\sigma }*u_{0})$.

### 2.3 The Third Step

In Eq. 9, gradient fidelity term is added to increase the smoothness of the image. However, whether it will impede the optimal solution has not been proved. If E(u)is the convex function, the optimal solution uniquely exists.

Theorem 1 if $\lambda_{1},\lambda_{2}>0,\lambda_{1}+\lambda_{2}=1,$

E(λ1u1+λ2u2)≤λ1E(u1)+λ2E(u2)

Proof:

(λ1u1+λ2u2−u0)2=[λ1(u1−u0)+λ2(u2−u0)]2=λ12u1−u02+λ22u2−u02+2λ2λ1(u1−u0)(u2−u0)=λ12u1−u02+λ22u2−u02−λ2λ1[(u1−u0)(u2−u0)]2≤λ12u1−u02+λ22u2−u02

Then,

(||∇(λ1u1+λ2u2)−∇(Gσ*u0)||)2=(||λ1∇u1−λ1∇(Gσ*u0)+λ2∇u2|−λ2∇(Gσ*u0)|)=λ12(||∇u1−∇(Gσ*u0)||)2+λ12(||∇u2−∇(Gσ*u0)||)2+2λ1λ2(∇u1−∇(Gσ*u0))(∇u2−∇(Gσ*u0))≤λ12(||∇u1−∇(Gσ*u0)||)2+λ12(||∇u2−∇(Gσ*u0)||)2

Therefore, 

E(λ1u1+λ2u2)≤λ1E(u1)+λ2E(u2)

An image can be regarded as the surface in two-dimensional space. The aim of using the gradient fidelity term is to keep the constraint of the continuity of the image topology. In the iterative computation, the gradient fidelity term maintains consistency of the original image and enhanced image, which can eliminate the loss of the image texture details and other features. Based on above analysis, Eq. 8 can be modified as:

(10) $$\frac{\partial u}{\partial t}=div\left(g\left(\left|\nabla G_{\sigma }*u\right|\right)\left|\nabla u\right|\right)-a\left(\nabla u-\nabla \left(\nabla G_{\sigma }*u\right)\right)$$

Where α is the weight coefficient, α>0.

### 2.4 The Fourth Step

 To increase detailed information, the strength coefficient is added in the spread model. The model is:

(11) $$\frac{\partial u_{t}(x,y)}{\partial t}=div\left(g\left(\left|\nabla G_{\sigma }*u\right|\right)\nabla u_{t}\right)+e^{w}\times u_{t}$$

Where w the strength coefficient, *div* is means divergence operator.

## 3. SIMULATION RESULTS

### 3.1 Enhancement Effect Analysis

To verify the effectiveness of IIEABPM, enhancement effect tests are done, as shown in Fig. (**3**).

In Fig. (**3**), the original images are (a1), (b1) and (c1), which are respectively hand bone image, angiocarpy image and rib image. (a2), (b2) and (c2) are enhanced images, accordingly. In the test, k_1_ =32, k_2_ =7, k_3_ =32, Δt=0.0001, n=25. From Fig. (**3**), we can see that enhanced images become clearer, and texture details have also been retained.

### 3.2 Performance Analysis

To further analyze the performance of IIEABPM, three tests are designed. 

**Fig. (3):**
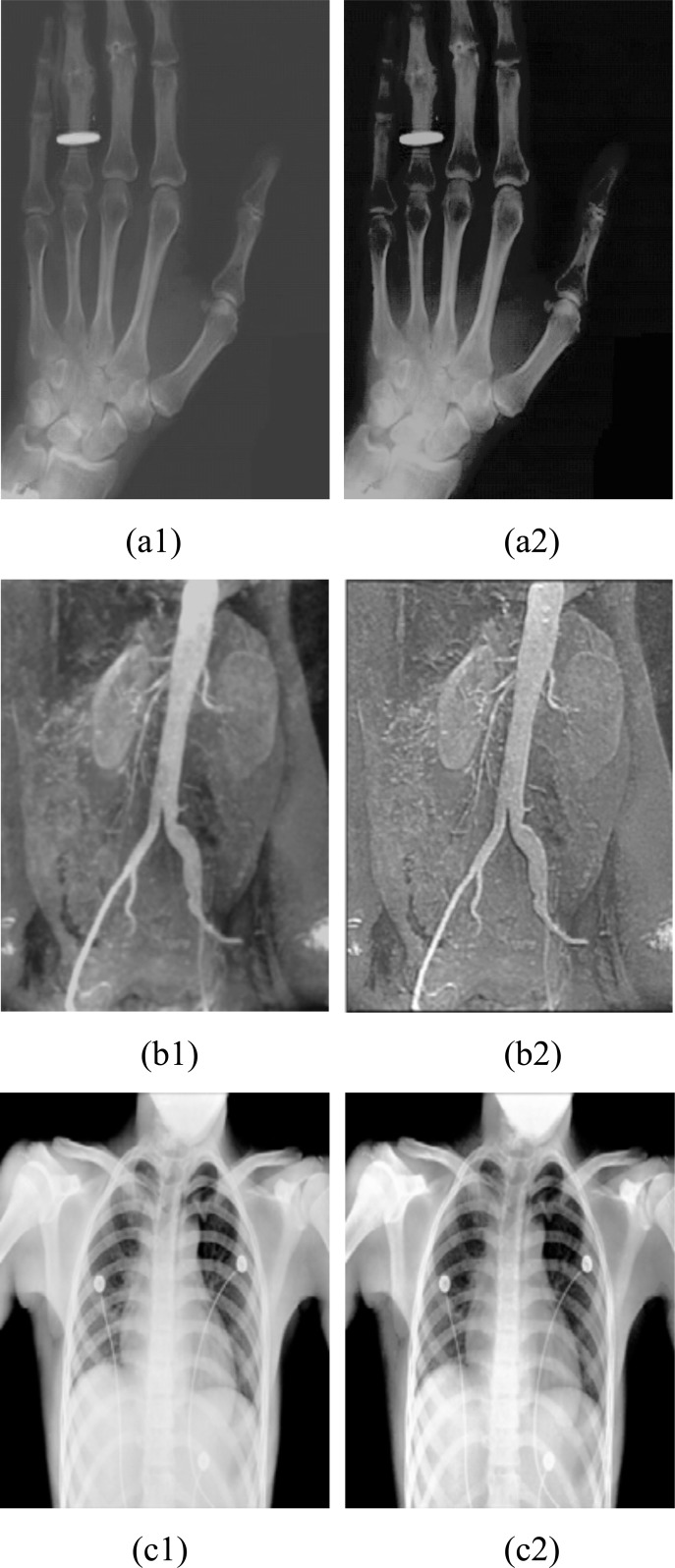
Enhancement effect.

In the first test, Fig. **3**(b1) is chosen as the subject, the performance of IEABPM and IIEABPM are compared in terms of the clarity, contrast, brightness and entropy. Fig. (**4**) gives the enhanced iamges, and Table **1** gives features of the enhaced images.

From Fig. (**4**) and Table **1**, we can see that the performance of IIEABPM is better than that of IEABPM.

**Fig. (4):**
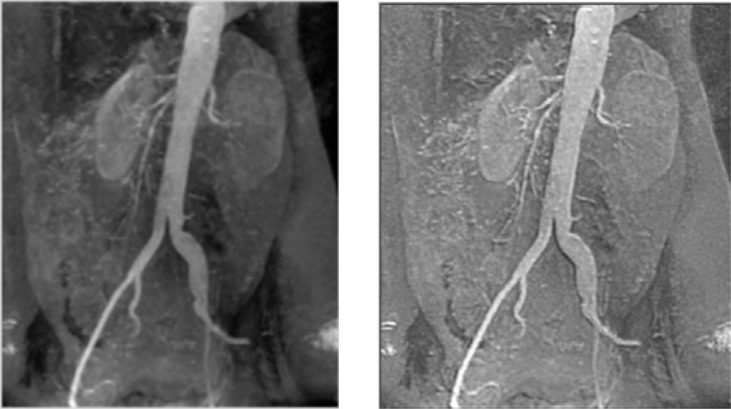
Enhanced images.

In the second test, Fig. **4**(**a**) with noise is chosen as the subject. The performance of image enhancement by the median filter, image enhancement by Gaussian filter, image enhancement by wavelet filter, IIEABPM and IEABPM are compared in the term of the enhancement effect, as shown in Fig. (**5**).

**Fig. (5):**
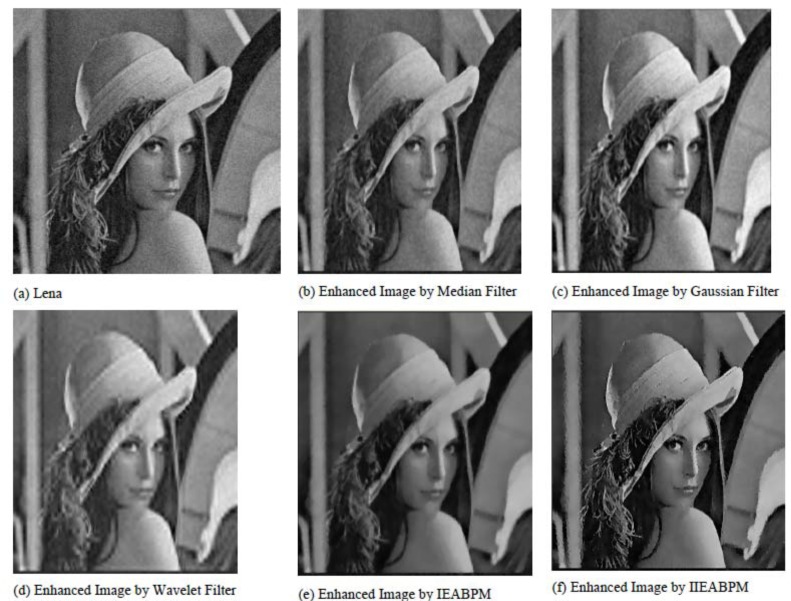
Compared results for Lena.

From Fig. (**5**), we can see that the performances of IEABPM and IIEABPM are better than the performances of the other. In Fig. **5**(**e**), many details are removed. However, in Fig. **5**(**f**), not only is the image noise is restrained, but also are details reserved.

In the third test, Fig. **3**(**a**) is chosen as the subject. The performance of image enhancement by the median filter, image enhancement by Gaussian filter, image enhancement by wavelet filter, IIEABPM and IEABPM are compared in the term of the enhancement effect, as shown in Fig. (**6**).

From Fig. (**6**), we can see that the performance of IIEABPM is the best.

## CONCLUSION

To solve the problems of IEABPM, the paper proposes IIEABPM. And simulation demonstrates that IIEABPM can effectively improve image clarity, image contrast, and iamge brightness.

## Figures and Tables

**Fig. (1):**
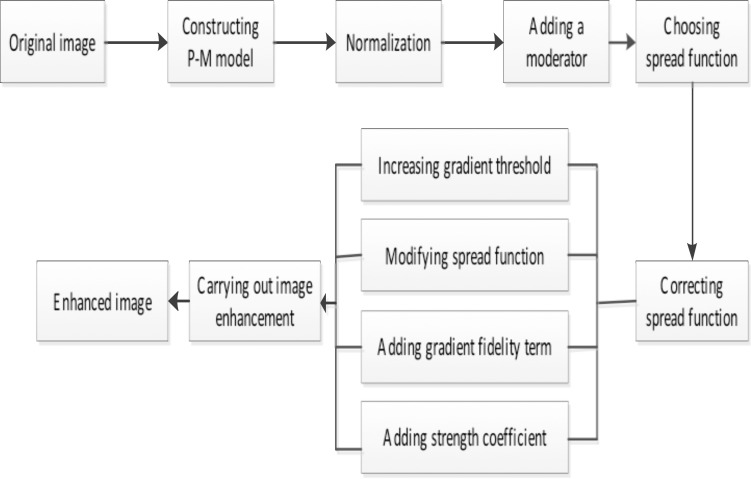
Flow chart of IIEABPM.

**Fig. (2):**
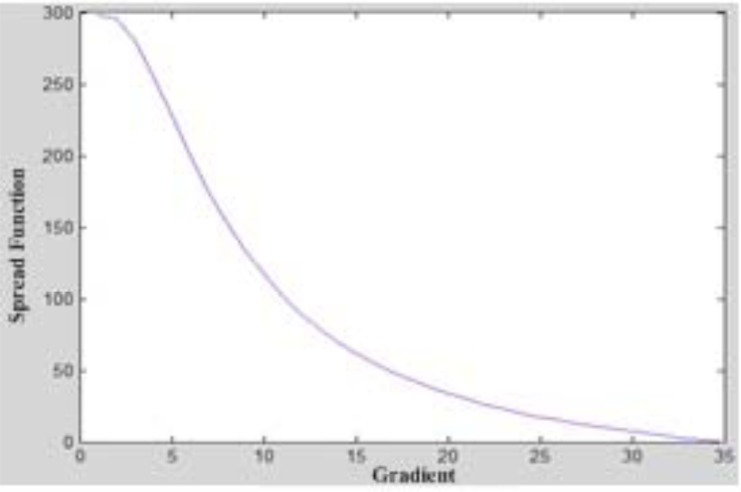
Gradient vs. Spread Function.

**Fig. (6):**
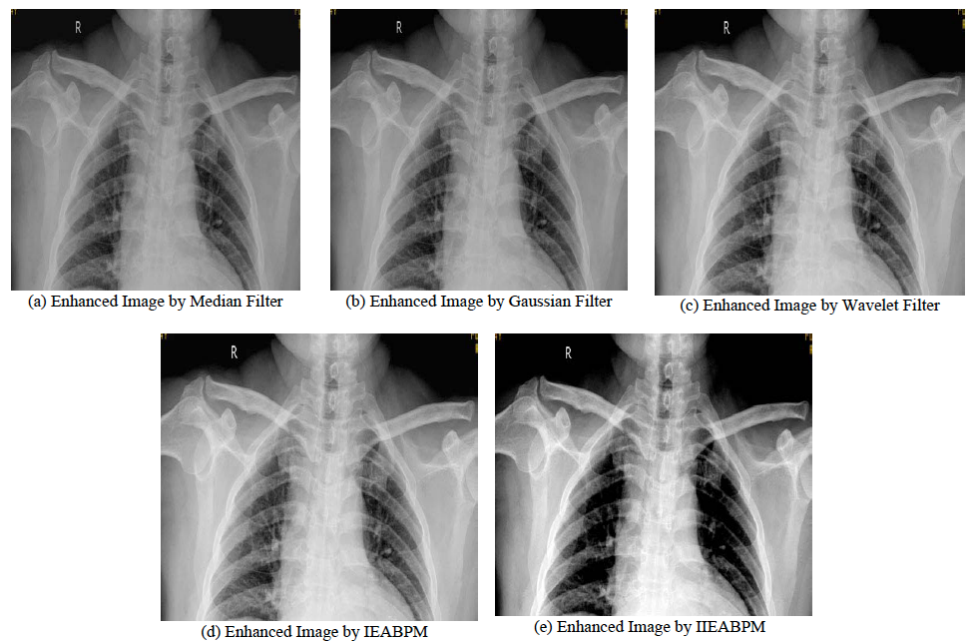
Compared results for rib image.

**Table 1.** Features of images.

	Brightness	Contrast	Entropy	Clarity
Original image	57.15	311.36	6.29	38.21
IEABPM	77.42	441.67	10.18	59.83
IIEABPM	79.26	487.82	14.01	73.85
